# Coherence and Coupling Functions Reveal Microvascular Impairment in Treated Hypertension

**DOI:** 10.3389/fphys.2017.00749

**Published:** 2017-10-13

**Authors:** Valentina Ticcinelli, Tomislav Stankovski, Dmytro Iatsenko, Alan Bernjak, Adam E. Bradbury, Andrew R. Gallagher, Peter B. M. Clarkson, Peter V. E. McClintock, Aneta Stefanovska

**Affiliations:** ^1^Physics Department, Lancaster University, Lancaster, United Kingdom; ^2^Faculty of Medicine, Saints Cyril and Methodius University of Skopje, Skopje, Macedonia; ^3^Deutsche Bank AG, London, United Kingdom; ^4^Department of Oncology & Metabolism, University of Sheffield, Sheffield, United Kingdom; ^5^Lancaster Medical Practice, Lancaster, United Kingdom; ^6^Department of Cardiology, Raigmore Hospital, Inverness, United Kingdom

**Keywords:** hypertension, cardiovascular regulation, aging, heart rate variability, microvascular blood flow oscillations, non-linear oscillator, coherence analysis, coupling functions

## Abstract

The complex interactions that give rise to heart rate variability (HRV) involve coupled physiological oscillators operating over a wide range of different frequencies and length-scales. Based on the premise that interactions are key to the functioning of complex systems, the time-dependent deterministic coupling parameters underlying cardiac, respiratory and vascular regulation have been investigated at both the central and microvascular levels. Hypertension was considered as an example of a globally altered state of the complex dynamics of the cardiovascular system. Its effects were established through analysis of simultaneous recordings of the electrocardiogram (ECG), respiratory effort, and microvascular blood flow [by laser Doppler flowmetry (LDF)]. The signals were analyzed by methods developed to capture time-dependent dynamics, including the wavelet transform, wavelet-based phase coherence, non-linear mode decomposition, and dynamical Bayesian inference, all of which can encompass the inherent frequency and coupling variability of living systems. Phases of oscillatory modes corresponding to the cardiac (around 1.0 Hz), respiratory (around 0.25 Hz), and vascular myogenic activities (around 0.1 Hz) were extracted and combined into two coupled networks describing the central and peripheral systems, respectively. The corresponding spectral powers and coupling functions were computed. The same measurements and analyses were performed for three groups of subjects: healthy young (Y group, 24.4 ± 3.4 y), healthy aged (A group, 71.1 ± 6.6 y), and aged treated hypertensive patients (ATH group, 70.3 ± 6.7 y). It was established that the degree of coherence between low-frequency oscillations near 0.1 Hz in blood flow and in HRV time series differs markedly between the groups, declining with age and nearly disappearing in treated hypertension. Comparing the two healthy groups it was found that the couplings to the cardiac rhythm from both respiration and vascular myogenic activity decrease significantly in aging. Comparing the data from A and ATH groups it was found that the coupling from the vascular myogenic activity is significantly weaker in treated hypertension subjects, implying that the mechanisms of microcirculation are not completely restored by current anti-hypertension medications.

## 1. Introduction

The complex variation in the human heart rate, well known as heart rate variability (HRV), has been studied extensively over the years (Billman, 2011). Although Hales (1733) had noted that the heart rate varied with respiration, known today as respiratory sinus arrhythmia (RSA), and Ludwig (1847) had already recorded RSA more than one-and-a-half centuries ago, the physiological origin of the processes involved in the frequency modulation of the heart rate is still widely disputed. Based on spectral analysis methods with linear frequency resolution, a ratio between low frequencies (usually linked with the activity of the sympathetic nervous system) and high frequencies (usually linked with parasympathetic activity) was proposed as a measure of health (Pagani et al., 1986; Malliani et al., 1991). This concept was subsequently disputed as greatly oversimplifying the complex non-linear interactions between the sympathetic and parasympathetic divisions of the autonomic nervous system (Eckberg, 1997) and it is now clear that the LF/HF ratio does not accurately measure cardiac sympatho-vagal balance (Billman, 2011).

Other approaches came from statistical physics and scaling properties (Amaral et al., 1998; Bernaola-Galván et al., 2001), multifractal properties (Ivanov et al., 1999), and 1/f spectra (Kobayashi and Musha, 1982; Ivanov et al., 2001) which were all proposed as ways of characterizing HRV. A reduction of variation was associated with sudden cardiac death and the Research Resource for Complex Physiologic Signals was created under the auspices of the National Center for Research Resources of the National Institutes of Health, intended to stimulate current research and new investigations in the study of cardiovascular and other complex biomedical signals (Goldberger et al., 2000).

Much of the HRV seems to be of deterministic origin, arising through a complicated interaction between physiological oscillations occurring on a wide range of different time scales (Stefanovska, 2007; Bashan et al., 2012). A promising approach, therefore, is to extract the deterministic features of the signals as far as possible, paying close attention to the non-linear and time-dependent dynamics of the parameters of cardiovascular regulation and in particular to the *coherence* and *coupling functions* between oscillatory components (Stefanovska and Bračič, 1999; Stefanovska et al., 2000; Smelyanskiy et al., 2005; Sheppard et al., 2012; Stankovski et al., 2012; Clemson and Stefanovska, 2014; Clemson et al., 2016). Moreover, we hypothesize that additional understanding might be gained by investigating the oscillatory components of signals measured at different sites of the cardiovascular system. In what follows we apply these approaches to gain insight into two particular states of the body that often co-exist in practice: aging and hypertension.

Because the functioning of the cardiovascular system is closely related to its efficiency in adapting to a time-varying environment, the couplings between its oscillating components could reveal its overall health. One aspect of aging is the progressive physiological weakening of the links that keep the cardiovascular system reactive and functional. This is why changes in the cardiovascular network with aging have been extensively investigated (Kelly et al., 1995; Jensen-Urstad et al., 1997; Agelink et al., 2001; Levy, 2001; Antelmi et al., 2004; Shiogai et al., 2010). As well as compromising the tone (Kelly et al., 1995) and elasticity (Levy, 2001) of the blood vessels, aging reduces HRV (Agelink et al., 2001; Antelmi et al., 2004; Shiogai et al., 2010) probably due to a weakening in couplings (Iatsenko et al., 2013).

Established hypertension can arise at any time of life, but predominantly occurs in the older age group, affecting about 40% of those over 25 (World Health Organisation, 2013). It is usually associated with an increase in the total peripheral resistance to blood flow, which contributes to high pressure while the cardiac output still remains normal. Many mechanisms have been proposed to account for the raised peripheral resistance. They include disturbances in renin-angiotensing system regulation, abnormalities of the sympathetic nervous system (Guyenet, 2006; McCurley et al., 2012), endothelial dysfunction (Taddei and Bruno, 2016), presence of specific genes expressed within the smooth muscle (Bai et al., 2013) and endothelial cells (Messaoudi et al., 2015), and vascular inflammation (Harvey et al., 2015). There is an associated loss of elasticity of the vessel walls accompanied by a reduction in their radii (Feihl et al., 2006). Hypertension is considered to be a major risk factor for heart disease, stroke and kidney failure and leads to premature death and disability (World Health Organisation, 2013). Currently available treatments that successfully reduce blood pressure also claim to revert the associated microvascular dysfunctions such as rarefaction and loss of reactivity (Taddei et al., 2000; Sörös et al., 2013).

Quite generally, the health and functionality of the human cardiovascular system can be assessed through the analysis of its associated signals, such as blood pressure and electrocardiogram (ECG). Heart rate variability (HRV, derived from the ECG) and blood pressure, analyzed in the time and frequency domains, have both diagnostic and prognostic value for essential hypertension (Verdecchia et al., 1994; Malik, 1996). The diagnostic and prognostic potential of skin blood flow, measured by laser-Doppler flowmetry (LDF), has taken longer to become generally appreciated (Rossi et al., 2011; Virdis et al., 2014). Several oscillatory components can be detected in LDF signals, of which the three fastest ones are (Bernardi et al., 1997; Stefanovska et al., 1999; Söderström et al., 2003; Stefanovska, 2007; Shiogai et al., 2010; Bernjak et al., 2012): cardiac (0.6–2 Hz, usually ≈ 1 Hz); respiratory (0.145–0.6 Hz, usually ≈ 0.25 Hz), and myogenic (0.052–0.145 Hz, usually ≈ 0.1 Hz). These oscillations are similar to those observed in HRV (Lotrič et al., 2000) and blood pressure (Stefanovska and Bračič, 1999). The 0.1 Hz oscillation corresponds to so-called Mayer waves (Julien, 2006) in blood pressure or so-called LF waves in HRV (Malik, 1996).

While the origins and nature of the cardiac and respiratory (known as HF in HRV) oscillations are generally agreed (Saul et al., 1991; Eckberg, 2003), the attribution of the mechanism underlying the 0.1 Hz oscillation differs, depending on whether it is being observed in cardiac or vascular activity. Studies of HRV and blood pressure variability emphasize the involvement of sympathetic nerve activity in oscillations around 0.1 Hz (Malpas, 2002; Julien, 2006), which are currently mainly attributed to time-delays in the baroreflex feedback loop. Qualitatively, changes in pressure are felt by baroreceptors that provide information continuously to the spinal cord. In response, appropriate sympathetic stimuli are generated and transmitted to all vascular beds and to the heart, aimed at maintaining the pressure within certain limits. Due to the finite response times, this sympathetic “correction” arrives after a delay, resulting in self-sustained oscillations. In contrast, studies of vascular dynamics mostly attribute 0.1 Hz oscillations to spontaneous movements of smooth muscle in the vessel wall, also known as myogenic activity, or Bayliss effect, or vasomotion. While the mechanism is not yet completely understood, it involves the opening and closing of ion channels in the endothelial and smooth muscle cells in the vessel walls (Aalkjaer and Nilsson, 2005) in response to changes in blood pressure. These vascular dynamical processes can be investigated through blood flow measurements.

**Figure 1:**
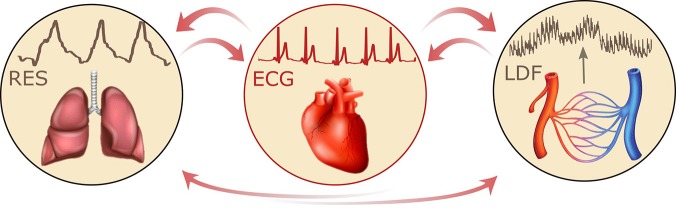
Schematic representation of the interactions between respiratory, cardiac and vascular activity, together with the corresponding recordings: respiratory effort signal (RES), electrocardiogram (ECG), and laser Doppler flowmetry (LDF).

Armed with the new method of time-localized wavelet phase coherence analysis (Sheppard et al., 2012) we have investigated the coherence between 0.1 Hz oscillations in HRV and LDF blood flow (also referred to as skin blood flow, or SBF) in order to establish how it changes with age and in treated hypertension. Furthermore, based on 30-min resting-state simultaneous recordings of the ECG, respiratory effort signal (RES), and LDF blood flow signal, the couplings between cardiac, respiratory, and microvascular activity were investigated, as indicated in Figure 1.

Non-linear mode decomposition (NMD) (Iatsenko et al., 2015) was used to extract the phases of the corresponding physiological modes. The instantaneous phase (frequency) was extracted individually around the subject's own characteristic rhythms, as found by wavelet transform. NMD was applied to extract the modes from the signals shown in Figure 1, both directly at source and from the LDF. Two networks of interacting oscillators were analyzed:

- *Central network*: cardiac from ECG (ϕ_*C*_), respiration from RES (ϕ_*R*_), and myogenic from LDF (ϕ_*m*_);
- *Peripheral network*: cardiac (ϕ_*c*_), respiration (ϕ_*r*_), and myogenic (ϕ_*m*_) all from LDF.

The first network describes the phase dynamics between the oscillations at their sources (indicated by subscript upper-case letters). Hence, the oscillations have different spatial origin. The second network describes how the couplings propagate into the blood flow (subscript lower-case letters). All three oscillations are detected in the same position in the microvasculature.

In this paper, we apply these advanced methods to provide a comprehensive analysis of oscillatory interactions. It enables us to investigate the effects of aging and treated hypertension on cardiovascular dynamics, at both the central and peripheral levels. First, however, as essential physiological background, we provide a more detailed description of the oscillations themselves.

### 1.1. Background: cardiovascular oscillations

The vascular network, the system of arteries, arterioles, capillaries, venules, and veins provides every cell of the human body with oxygen and nutrients, and carries away the waste metabolites. Cellular needs are dependent on the activity that the individual is performing, on the environmental conditions, on the health of the individual, and hence on time. Thus the cardiovascular system must be able to respond to time-dependent changes: centrally, the respiration and heart rates change significantly accordingly to need (Saul et al., 1991), and are coupled to each other (Figure 1). The modulation of heart rate by the frequency of respiration is known as RSA (Clynes, 1960). This modulation is easy to observe by simultaneous recordings of the ECG and respiratory effort and has frequently been reported to change with age (see e.g., Iatsenko et al., 2013) and cardiovascular diseases. However, as mentioned above, the mechanisms of physiological coupling that enable this modulation are not yet settled and remain a matter of intensive investigation.

Oscillations spanning a wide frequency range have also been observed in recordings from the microvasculature (Karstrup et al., 1989; Johnson, 1991; Bertuglia et al., 1994; Stefanovska et al., 1999). They occur at a number of characteristic frequencies, of which the three relevant to the present work are summarized in Table 1. We now consider them each individually.

**Table 1:** The oscillations analyzed.

**Oscillation**	**Characteristic** **frequency (Hz)**	**Range (Hz)**
Cardiac	1	0.6–2.0
Respiratory	0.25	0.145–0.6
Myogenic	0.1	0.052–0.145

#### 1.1.1. Cardiac and respiratory oscillations

The heart rhythmically pumps blood into the vascular system, and the corresponding oscillations propagate to the capillary bed, where they can be detected in skin blood flow by LDF (Bernardi et al., 1997; Rossi et al., 2006).

The respiratory (RES) activity of the lungs generates a wave of pressure that is propagating in the vascular network and can be detected even in the microvasculature using LDF (Hoffman et al., 1990; Bollinger et al., 1991; Stefanovska and Hožič, 2000).

#### 1.1.2. Vasomotion and myogenic oscillations

Vasomotion is the spontaneous oscillation in tone of blood vessel walls, independent of heart beat, innervation or respiration (Haddock and Hill, 2005). It consists of rhythmic oscillations in vessel diameter and has been detected both *in vitro* and *in vivo* (Aalkjaer et al., 2011). No specific frequency is currently associated with vasomotion, and the range reported, mostly based on visual inspection in the time domain, is quite wide spanning between 0.01 and 0.5 Hz. There are several reasons. The oscillations are not clock-like, but rather quasi-periodic. For the frequency content to be resolved in detail one needs long resting-state recordings (at least 30 min, or longer), and time-frequency spectral characterisation methods. The frequency content also varies from species to species, roughly scaling with heart rate and vessel size (Colantuoni et al., 1984b,a; Bertuglia et al., 1991; Stefanovska, 2007). The smooth muscle cells, endothelial cells and the sympathetic nerves innervating the vessels, are all involved in maintaining the vascular movement and each seems to manifest itself at a different frequency (Kvandal et al., 2006).

The existence of 0.1 Hz oscillations in vessel radius have frequently been reported in humans. These oscillations correspondingly modify blood flow to produce quasi-periodic fluctuations known as flowmotion (Schmidt et al., 1992). Using the wavelet transform, 0.1 Hz oscillations have been detected in signals measured by LDF (Kvernmo et al., 1998; Stefanovska et al., 1999; Kvandal et al., 2003; Söderström et al., 2003; Stefanovska, 2007). There is still no general agreement about their origin, despite extensive discussions in the literature. Some authors attribute these oscillations to the sympathetic nervous system (Stauss et al., 1998; Cevese et al., 2001) and have associated them with baroreceptor activity that modulates the frequency of the heart thereby controlling and stabilizing blood pressure. Others have concluded that the 0.1 Hz oscillation is caused directly by the spontaneous contractions of pressure-sensitive pacemaker cells within the smooth muscles of the arterial walls (Johnson, 1991; Söderström et al., 2003), and thus that it does not originate directly from the sympathetic system.

Studies on skin-flaps and under local or general anæsthesia have further elucidated the origin of these oscillations (Söderström et al., 2003; Landsverk et al., 2006, 2007). In these cases recordings have been made while sympathetic nerves reaching the vascular myocytes were either not existing, or temporarily blocked, and spontaneous myogenic (0.1 Hz) oscillations could be distinguished from the slower purely sympathetic oscillation (0.04 Hz). In what follows, we will therefore refer to the oscillations at around 0.1 Hz as *myogenic*.

Myogenic oscillations, whether spontaneously activated due to the smooth muscle cell ionic conductances, or stimulated by a sympathetic inflow, contribute to the regulation of vascular stiffness, which is of crucial importance in hypertension. Hence, their evaluation *in vivo* could help indicate the efficacy of different treatments.

## 2. Methods

### 2.1. Subjects

Three groups of subjects were investigated: 29 young healthy subjects (group Y, aged 24.4±3.4 years); 22 aged healthy subjects (group A, aged 71.1± 6.6 years); and 22 aged treated hypertensives (group ATH, aged 70.3 ± 6.7 years).

General data for all three groups of subjects are summarized in Table 2 including their systolic blood pressure (SBP). All subjects except ATH had SBP <150 mmHg and diastolic BP < 90 mmHg. All had body mass indices < 30, and skin temperature during recording >28.5°C. Clinically relevant information about the ATH group is given in Tables A1, A2 of the **Appendix I**. Informed consent was provided by all participants. The study was approved by the UK Northwest Research Ethics Committee.

**Table 2:** Age and blood pressure data of the three groups.

**Group**	**N**	**Age (y)**	**Min/Max (y)**	**SBP (mmHg)**
Y	29 (14F)	24.4 ± 3.4	18/29	118.2 ± 16.2
A	22 (13F)	71.1 ± 6.6	61/90	123.7 ± 12.5
ATH	22 (10F)	70.3 ± 6.7	59/84	138.8 ± 16.4

### 2.2. Signals and preprocessing

Signals were recorded for 30 min, with subjects relaxed and supine at room temperature 21 ± 1°C. The ECG was obtained from a bipolar precordial lead similar to the standard D2 lead. To maximize R-peak sharpness, electrodes were positioned on the right shoulder and in the fifth intercostal space in the left anterior axillary line. Respiratory effort was recorded using a belt encircling the subject's chest, fitted with a Biopac TSD201 Respiratory Effort Transducer (Biopac Systems Inc., CA, USA). Skin blood flow was measured by LDF, using a MoorLAB blood flow monitor with an MP1-V2 probe (Moor Instruments, Axminster, UK), with a near-infrared laser diode producing an output power of 1.0 mW at a wavelength of 780 nm. In the resting state, the concentration of red blood cells can be considered constant, and so a Doppler shift in the velocity signal provides a measure of microvascular flow. In what follows we will use “blood flow” for skin blood flow recorded in this way (also referred to as SBF). A flexible probe holder with probe was attached to the skin on the inside front of the right wrist (caput ulna) by a double-sided adhesive disk. The time constant of the flow monitor was set to 0.1 s. The signals were recorded simultaneously (16-bit A/D converter, sampling frequency 400 Hz) using a signal conditioning system (Cardiosignals, Institute Jožef Stefan, Slovenia).

The LDF signals contained no more than 1% of artifacts; the ECG recordings included fewer than 50 ectopic beats in total; and the breathing rates of all subjects lay within the normal physiological parameters for the respiratory frequency band (0.145–0.6 Hz).

The LDF signals were resampled to 40 Hz, and examined visually to check for movement artifacts, which were removed by interpolation with cubic Hermite polynomials.

R-R interval time-series (i.e., of beat-to-beat intervals, the reciprocal of HRV) were obtained from the R-peaks in the ECG signal (marked events method, with linear interpolation).

### 2.3. Analysis

We conducted a comprehensive analysis of the cardiovascular oscillations and their interactions. In doing so, we first investigated the *existence* and *strength* of the oscillations, then we *decomposed* and *extracted* the oscillations, after which we quantified their *coordination* and *coherence* so that, in the end, we were able to reconstruct the coupling functions describing the *interaction mechanisms*. The methods used are explained succinctly below.

#### 2.3.1. Existence and strength of the oscillations – use of the wavelet transform

**Figure 2:**
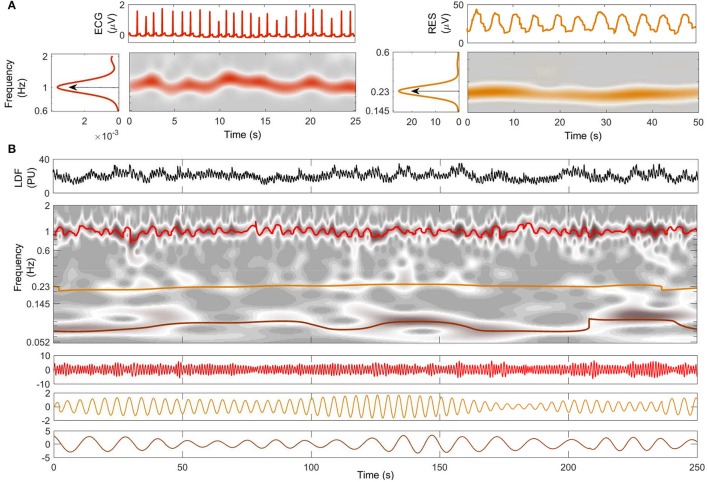
Decomposition into oscillatory modes. **(A)** Typical time windows of the signals, their wavelet transforms, and their averaged power spectra. The central frequency of each oscillation is shown for ECG (red) and respiration (orange). **(B)** A 250-s window of the LDF signal from the same subject is shown in the top panel. The time-frequency evolutions of the modes extracted by NMD (second panel) are indicated by color with heart-rate red, respiration orange, and myogenic brown. The time evolutions of the extracted modes are plotted below, with the same color-code.

The signals (examples in Figure 2) were first analyzed using the continuous wavelet transform (WT), which copes with the inherent non-stationarity and time-variability of physiological signals (Stefanovska et al., 1999). The WT also provides logarithmic frequency resolution (not achievable with a Fourier transform), thus yielding an appropriate representation of the low frequency spectral structure. Before applying the WT, signals were detrended by subtracting a 200 s moving average, and de-meaned. In this way, the frequency content was strongly attenuated below 0.005 Hz.

The continuous wavelet transform (WT) of a signal *s*(*t*) was used in the form

(1) $$\begin{array}{r} WT\left(\omega ,t\right)=\overset{\infty }{\underset{0}{\int }}\psi \left(\omega \left(u-t\right)\right)s\left(u\right)\omega \text{d}u, \end{array}$$

where ω denotes angular frequency, *t* is time, and $\psi \left(u\right)=\frac{1}{2\pi }\left(e^{i2\pi f_{0}u}-e^{\frac{{\left(2\pi f_{0}\right)}^{2}}{2}}\right)e^{\frac{-u^{2}}{2}}$ (with *i* the imaginary unit, a central frequency of *f*_0_ = 1, and ∫ ψ(*t*)*dt* = 0) is the complex Morlet wavelet.

The WT belongs to the family of time-frequency representations and contains both the phase and amplitude dynamics of the oscillatory components in the signal. With the normalizations of (1), the value of $|W_{s}\left(t,f\right){|}^{2}$, often called the normalized scalogram, can be regarded as the instantaneous power spectral estimate at each time *t*. We therefore refer to $|W_{s}\left(t,f\right){|}^{2}$ as the wavelet power, so that e.g., if one has *s*(*t*) = *A*cos(2π*t*) it will, at all times, have a peak of height *A*^2^ located at *f* = 1 Hz. The time-averaged wavelet power is quite similar to the usual power spectrum (estimated from the Fourier transform after smoothing in frequency or time). However, in the latter case the smoothing is performed with a constant window, leading to spectral resolution of the oscillations on the basis of their frequency difference, i.e., linear frequency resolution. Thus e.g., the spectral resolution between oscillations with periods of 10 and 100 s (0.1 and 0.01 Hz) will be almost the same as of that of oscillations with periods 10 and 5 s (0.1 and 0.2 Hz). In contrast, the WT has an adaptive window leading to logarithmic frequency resolution, distinguishing frequency components on the basis of the ratio of their frequencies (or periods), and thus yielding good resolution of the low-frequency spectrum.

Two typical windows of RES and ECG signals from a young subject are shown at the top of Figure 2A. The WT of each signal within the investigated frequency band is shown below its time series: a ridge corresponding to the characteristic frequencies of cardiac (left) and respiratory (right) activity clearly emerges in the time-frequency plane. The central frequency characterizing each subject's cardiac and respiration rhythms is then determined by the peak value of the time-averaged spectral information, shown by the arrows on the side panels of Figure 2A.

#### 2.3.2. Decomposition and extraction of oscillations – the use of non-linear mode decomposition

The recently-introduced method of NMD (Iatsenko et al., 2015) enables extraction of a time-variable oscillation by following the sequence of corresponding ridges in the wavelet transform plane (Iatsenko et al., 2016) and isolating the noise. Features of the method and related procedures used here include:

- The possibility of focusing the investigation of each mode on the appropriate section of the spectrum, i.e., on the subject-dependent central frequency detected by the WT.
- The use of ECG and RES time series as references for extracting cardiac and respiration oscillations from the LDF signals.
- The sequential subtraction of each decomposed mode from the original signal, before extracting the next one. This procedure excluded overlapping between the oscillations, thus enhancing the dynamical Bayesian inference (see below).

Figure 2B shows the results of NMD applied to the LDF signal from the same young subject as in part Figure 2A. The LDF time series is shown on the top in Figure 2B, and the corresponding WT is shown below it in gray-scale. The frequency evolution in time of the extracted modes is superimposed in color on the WT: red is used for cardiac, orange for respiration and brown for myogenic. Note that the colored lines follow the trend of the gray ridges and are centered around the frequencies determined in Figure 2A. The time series corresponding to the modes are illustrated in the three panels below the WT in Figure 2B, following the same color code as before. Both frequency and amplitude modulation, consistent with the WT ridges, are evident in this representation.

#### 2.3.3. Coordination of oscillations – the use of wavelet phase coherence

Frequency-resolved phase coherence is a useful technique for studying the phase relations and coordination of the oscillations (Mormann et al., 2000; Bandrivskyy et al., 2004; Sheppard et al., 2012; Xie et al., 2017). The phase coherence between the two signals *s*_1, 2_(*t*) is determined through their WTs as

(2) $$\begin{array}{r} WPC\left(f\right)=\sqrt{{\langle \sin\left(\Delta_{\phi }\left(f\right)\right)\rangle }^{2}+{\langle \cos\left(\Delta_{\phi }\left(f\right)\right)\rangle }^{2}}, \end{array}$$

with Δ_ϕ_(*f*) equal to the difference of the WT angles of *s*_1_(*t*) and *s*_2_(*t*) at the frequency *f* and all times. It reflects the extent to which the phases (and thus the underlying activities) of these signals at frequency *f* are correlated. Unlike the usual coherence measures, wavelet phase coherence takes no account of the amplitude dynamics of the signals. This is appropriate because the relationships between the amplitudes of common physiological oscillations in different signals can be complicated and non-linear, but in all cases the relationship between their phases remains the same (up to the constant phase shift).

*Time-localized coherence*. To reveal the evolution of coherence in time, one can calculate it in a sliding window, in which case it is called time-localized coherence (Sheppard et al., 2012). To establish the appropriate amount of information for a reliable coherence measure at each frequency, we use adaptive windows of time length *nc*/*f*, which thus contain the chosen number of *nc* cycles at each frequency. We use *nc* = 50 for the time-localized coherence presented in Figure 5C.

#### 2.3.4. Interaction mechanisms – coupling functions through the use of bayesian inference

Modeling the data with coupled phase oscillators (Kuramoto, 1984), we apply dynamical Bayesian inference (DBI) to extract the optimal set of parameters describing the model. The method is capable of isolating the noise, and following the time-varying behavior typical of living systems (Duggento et al., 2008; Stankovski et al., 2012; Wilting and Lehnertz, 2015). By decomposing the system into a set of interacting phase oscillators, it is possible to isolate the specific influence of each oscillator on the others, in order to generate the observed behavior of the system, i.e., the effective coupling (Kralemann et al., 2013; Stankovski et al., 2014a).

The dynamical mechanism of interaction between a pair of oscillators can be described visually by the form of the corresponding coupling function (Kralemann et al., 2011; Stankovski et al., 2012, 2015, 2017a). To facilitate comparisons between coupling functions, two quantities have been calculated: (i) the *coupling strength* (σ) (7), based on the Euclidean norm of the coupling coefficients (Kralemann et al., 2013; Stankovski et al., 2015); and (ii) the *maximal polar similarity* (ρ) (Stankovski et al., 2017b) (9). The latter index, introduced here, is based on bi-dimensional correlation, and can thus capture specific features of the coupling functions by quantifying their morphological similarities (Kralemann et al., 2013; Stankovski et al., 2015) and phase shift.

Numerous methods exist for the inference of interactions between oscillators (Rosenblum and Pikovsky, 2001; Varela et al., 2001; Paluš and Stefanovska, 2003; Bahraminasab et al., 2008; Jamšek et al., 2010; Jirsa and Müller, 2013). Among them, dynamical Bayesian inference (DBI) (Smelyanskiy et al., 2005; von Toussaint, 2011; Stankovski et al., 2012) has the power to provide information, not only about the presence of an interaction, but also about its underlying mechanisms. In this mathematical context, *mechanism* is defined by the functional form which specifies the rule and process through which the input values are translated into output values, i.e., for a particular system it prescribes how the input influence from a second system gets translated into consequences in the output of the first system.

To tackle the inverse problem of determining coupling connections from a measured signal, the system is modeled as a network of *N* coupled phase oscillators (Kuramoto, 1984; Pikovsky et al., 2001). The system of *N* stochastic differential equations subject to noise has time-varying parameters, and it is defined as:

(3) $$\begin{array}{r} \dot{\phi_{i}}\left(t\right)=\omega_{i}\left(t\right)+q_{i}\left(\phi_{i},\phi_{j},\phi_{k},\ldots ,\phi_{N},t\right)+\xi_{i}\left(t\right) \end{array}$$

with *i* = 1, …, *N*, where the instantaneous frequency ${\dot{\phi }}_{i}$ of each oscillator is determined by the combination of its natural frequency ω_*i*_ and a function *q*_*i*_ of all the *N* oscillators' phases ϕ_1, …, *N*_ representing the coupling configuration. The stochastic part is modeled by the Gaussian white noise ξ_*i*_. The deterministic periodic part of (3) can be Fourier-decomposed into a sum of base functions Φ_*k*_ = exp[*ı*(*k*_1_ϕ_1_ + *k*_2_ϕ_2_ + …+*k*_*N*_ϕ_*N*_)] (Kralemann et al., 2011; Duggento et al., 2012), characterized by the time-varying bank of parameters $c_{k}^{\left(i\right)}$:

(4) $$\begin{array}{r} \dot{\phi_{i}}\left(t\right)=\overset{K}{\underset{k=-K}{\sum }}c_{k}^{\left(i\right)}\Phi_{k}\left(\phi_{1},\phi_{2},\ldots ,\phi_{n}\right)+\xi_{i}\left(t\right), \end{array}$$

where *K* is the order of the Fourier expansion. In this study it was set *K* = 2. Starting from the phase dynamics extracted from the time-series, the aim is to compute the set of parameters $M=\left\{c_{k}^{\left(i\right)},D_{r,s}\right\}$ which completely describes the couplings ($c_{k}^{\left(i\right)}$) and the noise (*D*_*r, s*_).

Bayes' theorem (Bayes, 1763) allows one to obtain the *posterior* density $p_{X}\left(M|X\right)$ of the unknown matrix of parameters M from X, given a *prior* density $p_{\text{prior}}\left(M\right)$ (based on observations and representing previous knowledge of the unknown parameters), by building a *likelihood* function ℓ(X|M):

$$\begin{array}{r} p_{X}\left(M|X\right)=\frac{\ell \left(X|M\right)p_{\text{prior}}\left(M\right)}{\int \ell \left(X|M\right)p_{\text{prior}}\left(M\right)dM}. \end{array}$$

The likelihood function is computed through the stochastic integral of the noise term over time, leading to the minus log-likelihood function S=-ln ℓ(X|M) expressed as:

(5) $$\begin{array}{l} \begin{array}{cc} S & =\frac{L}{2}\ln\;|\text{D}|+\frac{h}{2}\overset{L-1}{\underset{l\;=\;0}{\sum }}{\text{c}}_{k}\frac{\partial {\text{Φ}}_{k}\left(\phi_{\cdot ,l}\right)}{\partial \text{ϕ}}+\\ +{\left[{\dot{\text{ϕ}}}_{l}-{\text{c}}_{k}{\text{Φ}}_{k}\left({\text{ϕ}}_{\cdot ,l}^{*}\right)\right]}^{T}\left({\text{D}}^{-1}\right)\left[{\dot{\text{ϕ}}}_{l}-{\text{c}}_{k}{\text{Φ}}_{k}\left({\text{ϕ}}_{\cdot ,l}^{*}\right)\right], \end{array} \end{array}$$

where summation over the repeated indices *k* is implicit, and the dot index in **ϕ**_·_ is substituted with the relevant index.

Assuming that the prior probability of parameters M is a multivariate normal distribution, and taking into account the quadratic form of the log-likelihood (5), the posterior probability will also be a multivariate normal distribution. This particular distribution for the parameters **c**, with mean $\bar{\text{c}}$, and covariance matrix $\sum_{\text{prior}}\equiv \Xi^{-1}{}_{prior}$, can be used to calculate recursively the stationary point of *S* only with the following four equations:

(6) $$\begin{array}{l} \begin{array}{cc} \text{D} & =\frac{h}{L}{\left({\dot{\text{ϕ}}}_{l}-{\text{c}}_{k}{\text{Φ}}_{k}\left({\text{ϕ}}_{\cdot ,l}^{*}\right)\right)}^{T}\left({\dot{\text{ϕ}}}_{l}-{\text{c}}_{k}{\text{Φ}}_{k}\left({\text{ϕ}}_{\cdot ,l}^{*}\right)\right),\\ {\text{r}}_{w} & ={\left({\text{Ξ}}_{\text{prior}}\right)}_{kw}{\text{c}}_{w}+h{\text{Φ}}_{k}\left({\text{ϕ}}_{\cdot ,l}^{*}\right)\left({\text{D}}^{-1}\right){\dot{\text{ϕ}}}_{l}+\\ -\frac{h}{2}\frac{\partial {\text{Φ}}_{k}\left(\phi_{\cdot ,l}\right)}{\partial \text{ϕ}},\\ {\text{Ξ}}_{kw} & ={\left({\text{Ξ}}_{\text{prior}}\right)}_{kw}+h{\text{Φ}}_{k}\left({\text{ϕ}}_{\cdot ,l}^{*}\right)\left({\text{D}}^{-1}\right){\text{Φ}}_{w}\left({\text{ϕ}}_{\cdot ,l}^{*}\right),\\ {\text{c}}_{k} & ={\left({\text{Ξ}}^{-1}\right)}_{kw}{\text{r}}_{w}, \end{array} \end{array}$$

where the summations over *l* = 1, …, *L*, and over the repeated indices *k* and *w*, is implicit. This inference technique is applied to the information provided by a stream of sequential blocks coming from the time-series and a special procedure is used for inferring time-varying dynamics. A tutorial about the practical implementation of dynamical Bayesian inference, including programming and software codes, is available (Stankovski et al., 2014b; NBP-Lancaster, 2016).

*Coupling strength:* The strength σ_*i, j*_ of the coupling from the oscillator *i* to *j* is defined as the Euclidean norm of the inferred parameters from the phase dynamics:

(7) $$\sigma_{i,j}=\sqrt{\sum_{k\;=\;-K}^{K}(c_{k}^{\left(i:j\right)}{)}^{2}},$$

where the parameters are defined as in Equation (4). It gives an overall estimate of the amount of influence that the phase of the oscillator *i* exerts on the frequency of the oscillator *j*.

*Polar similarity:* By calculating the correlation between coupling functions, one can quantify their similarity (Kralemann et al., 2013). Here we extend this concept, by computing the correlation of a coupling function *q* with a bank of numerically generated forms *Q* having specific shape features, in order to determine which of those features is predominant in *q*. The similarity modulus is defined as

(8) $$\begin{array}{l} |\rho_{q}|=\frac{\langle \tilde{q}\;{\tilde{Q}}_{\theta }\rangle }{\left|\tilde{q}|\;|{\tilde{Q}}_{\theta }\right|}\times 100, \end{array}$$

where the angular brackets denote averaging over the 2π × 2π phase grid and the tilde ~ denotes the deviation from the mean. Values of |ρ| range from 0 to 100 and are expressed as percentages. We generate coupling functions numerically with a shape which results from a unidirectional direct coupling of the slower oscillator to the faster, phase-shifted by an angle θ along the ϕ_1_ axis. Thus, the numerical form *Q*_θ_ generating the highest |ρ| carries dual information: the extent of the similarity (described by |ρ| itself) and the corresponding phase-shift angle θ generating it, denoted by 〈ρ〉. A natural way to represent this information is by indicating it on the complex plane by the *maximal polar similarity index*, defined as:

(9) $$\begin{array}{l} \rho_{q}=|\rho_{q}|e^{i\langle \rho_{q}\rangle }, \end{array}$$

where *i* is the imaginary unit.

**Figure 3:**
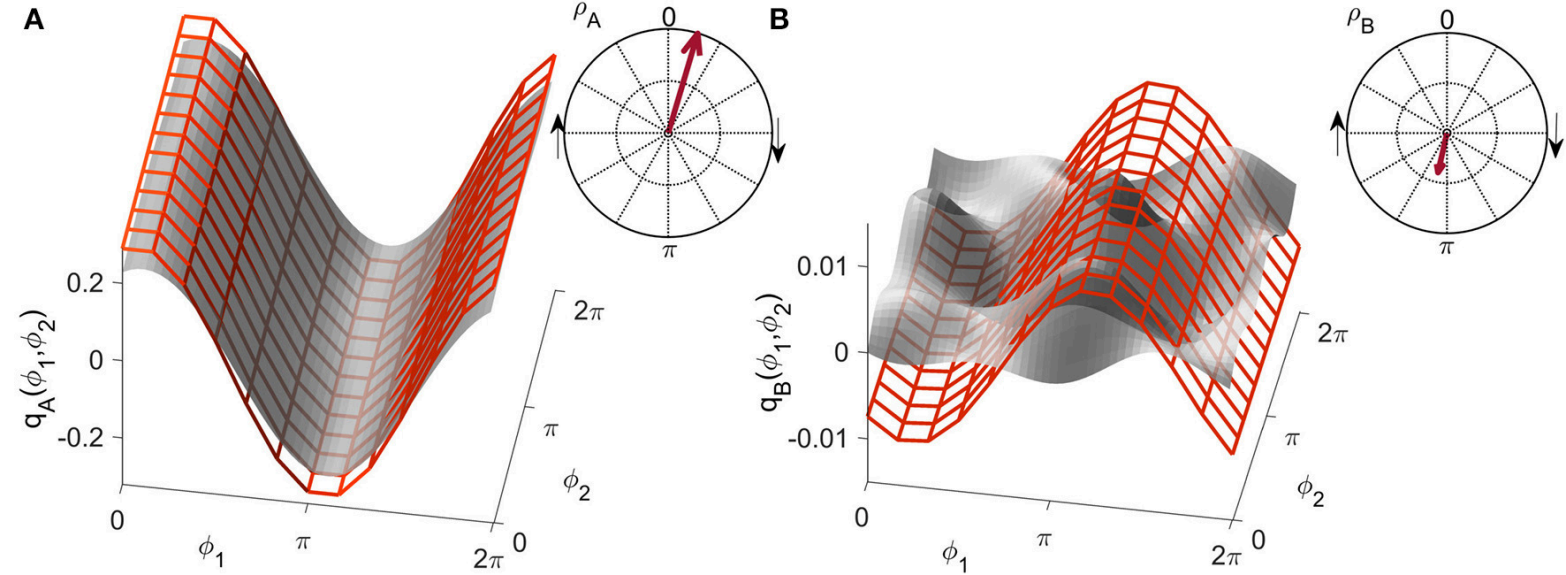
Examples of the similarity index for **(A)** high and **(B)** low similarity. The form obtained numerically from a unidirectionally coupled system, shown with a red grid, is shifted along the coupling function obtained from measured data, shown in gray, to detect the highest similarity modulus $\bar{\left|\rho \right|}$. The arrow in the polar plot has a modulus equal to $\bar{\left|\rho \right|}$ and a phase $\langle \bar{\rho }\rangle$ corresponding to the phase-shift of the red grid.

The meaning of this parameter is illustrated by the two examples in Figure 3, where the gray forms have been selected for generating very high and very low similarity indices, respectively. The superimposed red grid shows the most similar numerical form detected by the method. The arrows in the polar plots correspond to the polar similarity indices: the moduli of the arrows quantify the degree of overlap, while the angle indicates the phase of the positive maximum in the numerically generated function.

### 2.4. Statistical analysis and surrogates

An unpaired two-sided Wilcoxon rank sum test was used for comparisons between groups, and statistical significance was set at *p* < 0.05.

Because the phase coherence will generally be non-zero, even for completely unrelated oscillations, one needs to fix a threshold (significance level) above which coherence can be regarded as indicating genuine interdependence. For standard spectral coherence it is often set to 0.5, but such a threshold does not take into account the possibility of bias. We use the more reliable and accurate approach of applying a surrogate test. At each frequency we estimate the coherence threshold as being 95% of the highest value of the 300 coherences calculated between R-R intervals and skin blood flow taken from different subjects. Such signals are by definition completely independent, thus providing reliable estimates that take account of possible computational or methodological bias. Generally one is interested only in coherence above the threshold, so we consider an effective coherence defined as the actual coherence minus the calculated coherence significance level.

Inter-subject surrogate analysis (Toledo et al., 2002; Iatsenko et al., 2013) was also used to validate the results for coupling functions. The same central and peripheral networks were built for 200 combinations of randomly chosen inter-group subjects (i.e., cardiac from subject A, respiration from subject B, myogenic from subject C). Each such combination is therefore composed of mutually independent signals, but preserves the statistical characteristics of the original networks. This technique allows us to identify the significance of the results by comparison with the randomly created outputs, excluding from consideration as irrelevant any result that is lower than would be given by chance or which might arise through bias.

## 3. Results

### 3.1. Spectral power

#### 3.1.1. Spectral power in RR fluctuations

Fluctuations in R-R intervals at all frequencies decline markedly with age (not shown), in agreement with earlier work (Agelink et al., 2001; Antelmi et al., 2004; Shiogai et al., 2010). No statistically significant difference between the A and ATH groups was noticed, although the spectral power below 0.1 Hz tended to be lower for ATH.

#### 3.1.2. Spectral power in blood flow

**Figure 4:**
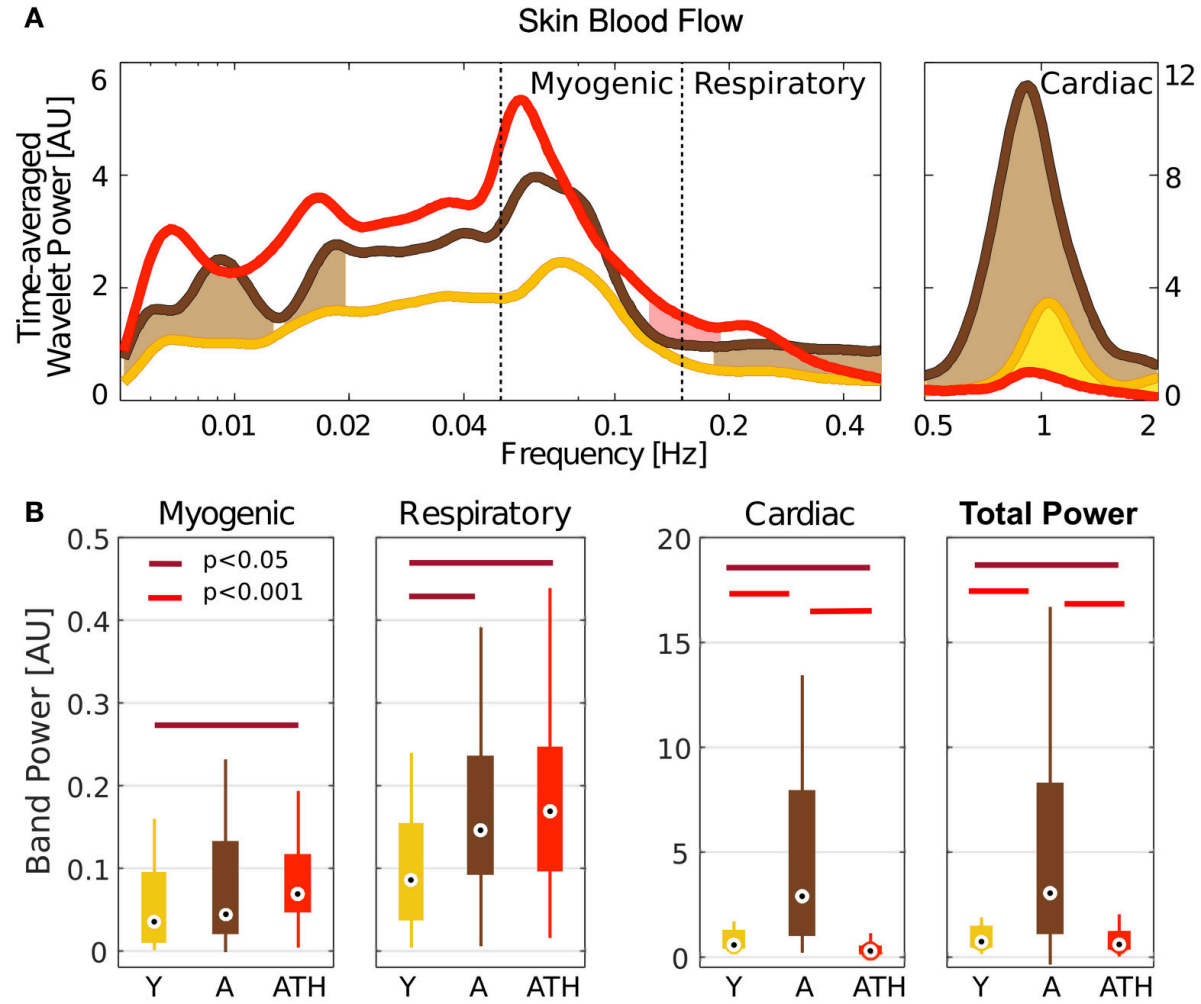
Wavelet power: **(A)** Time-averaged wavelet power of blood flow, means over groups. Brown shading indicates significance between A and Y, red shading between ATH and A, and yellow shading between Y and ATH. **(B)** Box-plots showing the cardiac, respiration and myogenic oscillations and the total power in the LDF signal within these three intervals. The Y group is represented in gold, A in brown, and ATH in red.

Very slow (<0.02 Hz), respiratory and cardiac oscillations in blood flow increase significantly with age (Figure 4A). While there is almost no difference in blood flow spectral power below 0.5 Hz between the A and ATH groups, there is a striking difference between them in the cardiac frequency range, showing that cardiac pulses in the ATH group are weaker than in the A group.

The box-plots in Figure 4B compare the power spectral content within the bands investigated for different groups: Y group is represented in gold, A in brown, and ATH in red. Group A was found to have strikingly stronger LDF cardiac oscillations than either the Y or ATH groups (*p* < 0.001). These oscillations carry most of the total power. For the respiration band, values of the power are less widely-separated, yet are significantly lower in the Y group (*p* < 0.05). A similar pattern was found within the myogenic band, with statistically significant differences only for the Y-ATH comparison.

### 3.2. Coherence between fluctuations in R-R variability and blood flow

**Figure 5:**
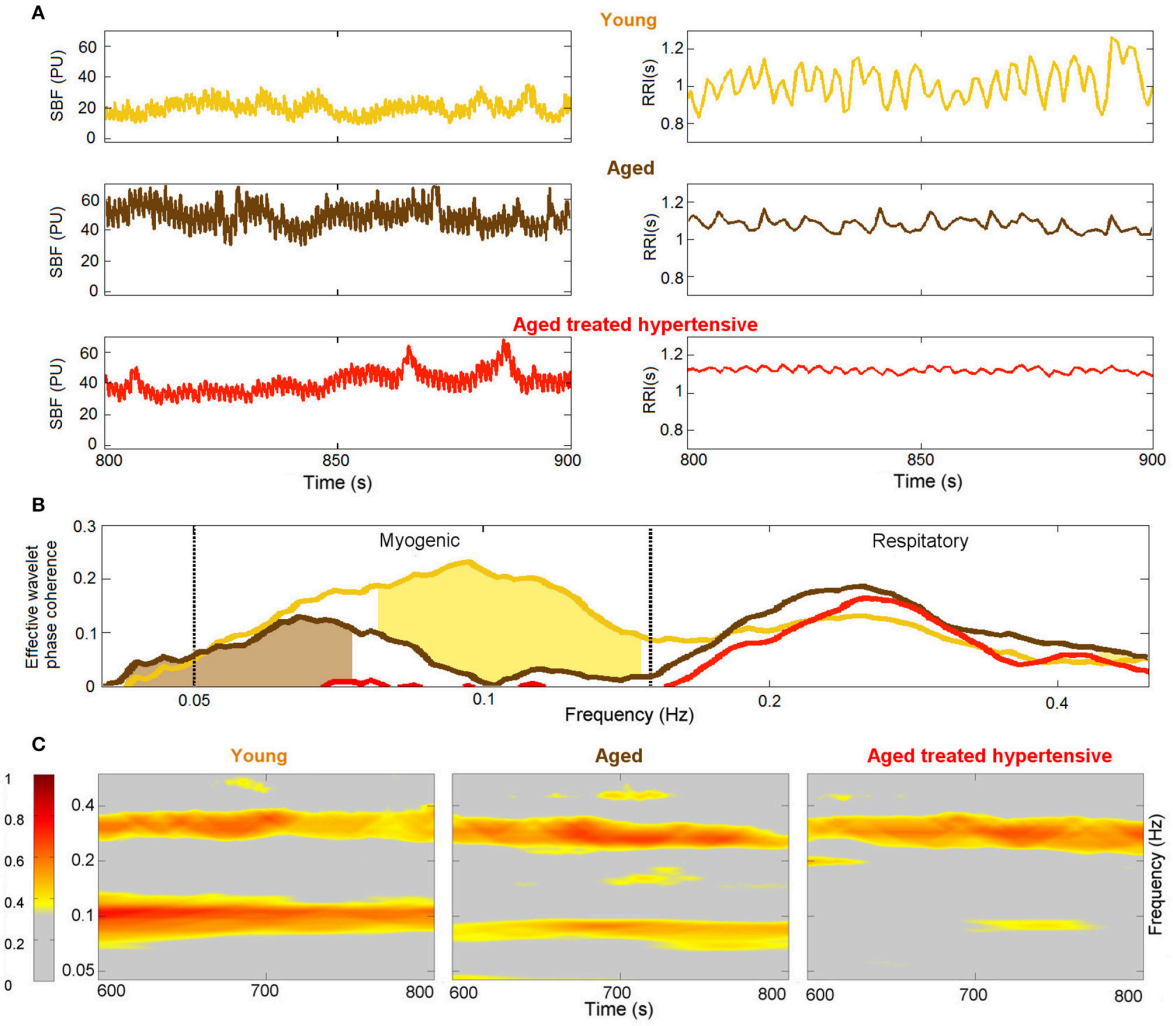
Phase coherence: **(A)** Typical SBF and R-R interval (RRI) signals from each group of subjects. PU – perfusion units; **(B)** Wavelet phase coherence (minus surrogate thresholds) between R-R intervals and SBF, mean over groups, where gold shading indicates significant difference between the Y and A groups and brown shading – between the A and ATH groups; **(C)** Time-localized wavelet phase coherence for individuals typical of the three subject groups. Note how the coherence within the myogenic interval is diminished almost to vanishing point in the ATH group.

**Figure A3:**
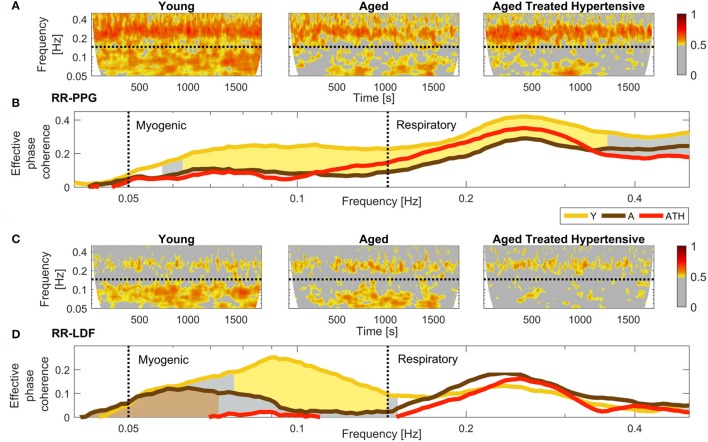
Comparison of RR-PPG, **(A,B)** with the corresponding RR-LDF, **(C,D)** phase coherences. Panels **(A,C)** show time-localized phase coherence obtained as a average for each group. Panels **(B,D)** show the time-averaged wavelet phase coherence (minus surrogate thresholds), and then averaged over groups; gold shading indicates significant difference between the Y and A groups, gray shading between Y and ATH, and brown shading between the A and ATH groups. Note how the coherence within the myogenic interval is higher in the Y than in the A and ATH groups in **(B)** and that it is diminished almost to vanishing point in the ATH group in **(D)**.

The results plotted in Figure 5B show that there is significant coherence (i.e., above the significance threshold) between skin blood flow and RR intervals in both the respiratory and myogenic intervals. However, only within the myogenic interval does the coherence differ between the groups, declining with age and nearly disappearing in treated hypertension. Figure 5C presents typical examples of time-localized coherence between R-R intervals and blood flow (Sheppard et al., 2012). It not only shows that the coherence can be stable in time, but also illustrates the decrease of coherence with age and its virtual disappearance in the ATH group. In particular, 28/29 Y and 18/22 A subjects, but only 11/22 ATH subjects, had significant coherence in myogenic interval. There were no significant gender differences in the effective coherence within each group (not shown). Time evolution of the average wavelet coherence for each of the three groups is shown in the **Appendix II**, **Figures A3C,D**. The coherence between R-R interval time series and the finger pulse plethismography (PPG) signal is also discussed in **Appendix II** and shown in **Figures A3A,B**. The (PPG) signal provides a measure of changes in arterial volume proportional to changes in arterial blood pressure.

### 3.3. Coupling functions

**Figure 6:**
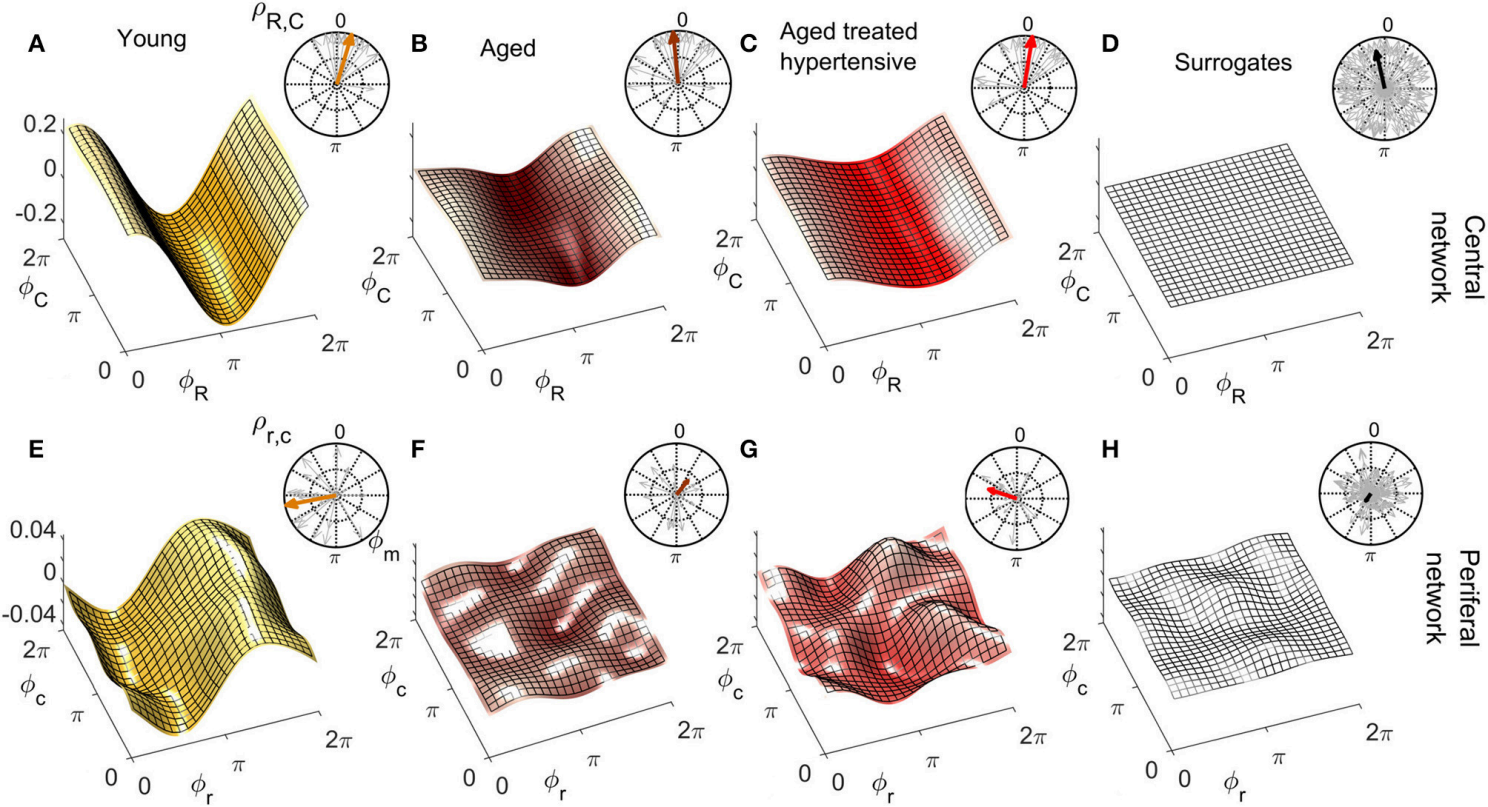
Group-averaged coupling functions in the central network (top row) compared with equivalent results from the peripheral network (bottom row). In each case the color coding is: Y (gold), A (brown), ATH (red), and surrogates (gray). **(A–C)** Show the coupling functions $q_{C}^{R,C}$ between the phases of centrally measured respiratory ϕ_*R*_ and cardiac ϕ_*C*_ oscillations, and **(E–G)** Show the equivalent quantity $q_{c}^{r,c}$ between the phase of respiratory ϕ_*r*_ and cardiac ϕ_*c*_ oscillations in the peripheral network. **(D,H)** show the surrogate coupling functions computed to check the validity of the results presented in each row. The polar plot in the top-right corner of each figure indicates the similarity index ρ for the average form (colored arrow) and for the individual subjects (gray arrows). Note how, with aging, the forms lose amplitude in the central network and resemble the variability of surrogates in the peripheral network.

**Figure 7:**
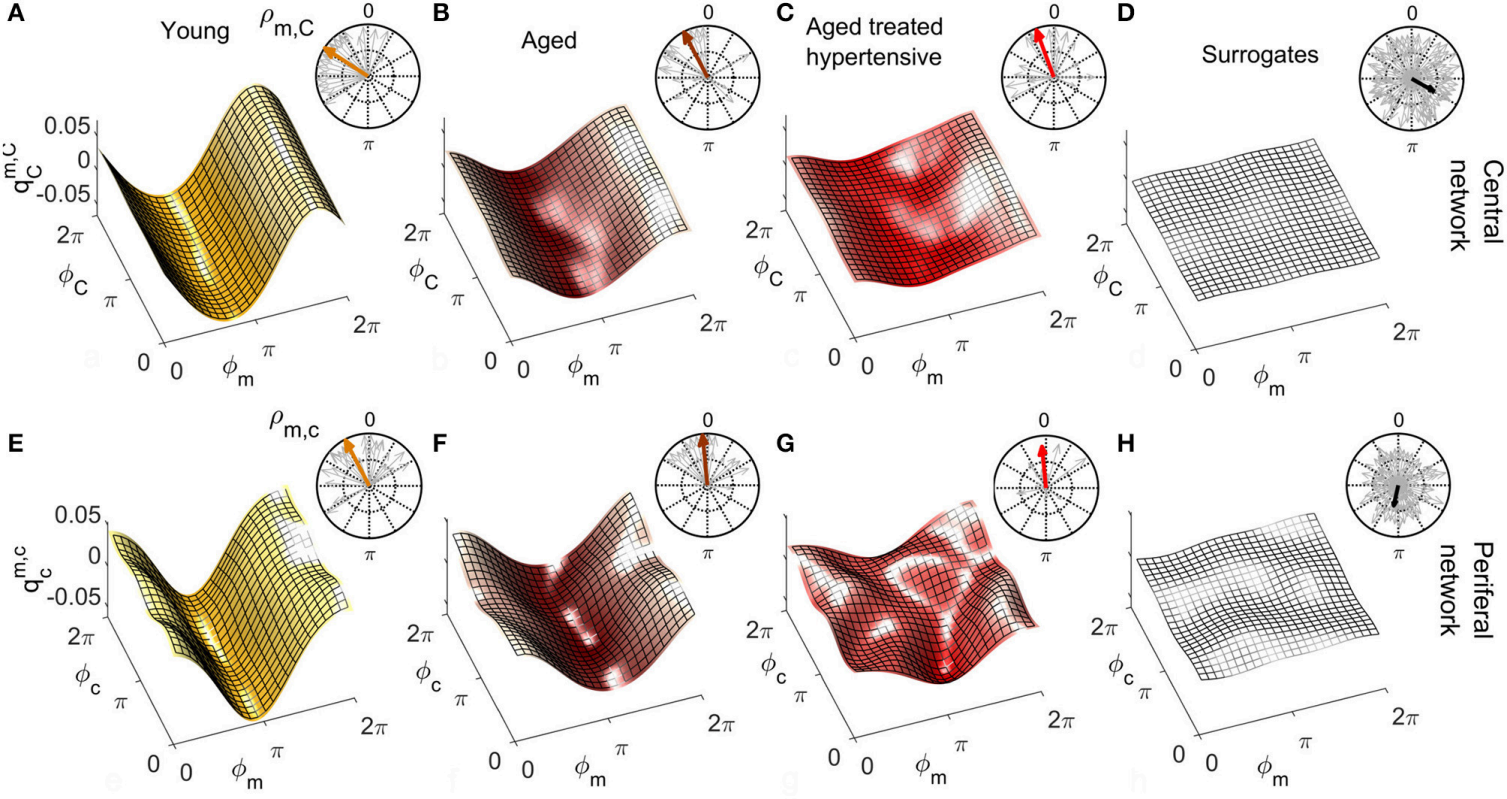
As in Figure 6 except that **(A–C)** represent the coupling functions $q_{C}^{m,C}$ between the phase of myogenic ϕ_*m*_ and cardiac ϕ_*C*_ oscillations in the central network and **(E–G)** show $q_{c}^{m,c}$ between the phases of myogenic ϕ_*m*_ and cardiac ϕ_*c*_ oscillations in the peripheral network. Plots **(D,H)** are from the corresponding surrogates. Again, the forms lose amplitude with aging in the central network, and with hypertension, resemble the variability of surrogates in the peripheral network.

**Figure 8:**
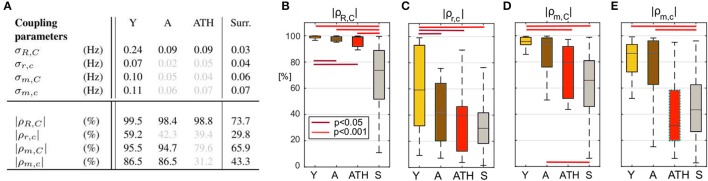
Statistics for coupling parameters. **(A)** Table showing the median values for the coupling strength σ and modulus of similarity |ρ| for groups of Y, A, ATH subjects, and surrogates, with median values not significantly different from surrogates are shown in gray. Box-plots picturing the distributions of the similarity modulus |ρ| within each group, using the same color map as Figure 4. The relation between respiration and cardiac for the **(B)** central and **(C)** peripheral networks and of myogenic and cardiac for the **(D)** central and **(E)** peripheral networks.

Figures 6, 7 show the group-averaged forms of coupling for the Y (Figures 6A,E, 7A,E), A (Figures 6B,F, 7B,F), ATH subjects (Figures 6C,G, 7C,G), and surrogates (Figures 6D,H, 7D,H). In each case, the first row shows the coupling detected from the centrally extracted modes, while the second row shows the results from the peripheral network. The polar graph in the top-right corner of each plot indicates the polar similarity index ρ for each subject (thin arrows in gray) and $\bar{\rho }$ for the inter-subject average (thick colored arrow). Values of the median similarity modulus $\bar{\left|\rho \right|}$ and strength σ for each group are listed in Figure 8A.

The group distribution of similarity $\bar{\left|\rho \right|}$ is shown by the box-plots in Figures 8B–E. The lines over the boxes indicate the statistically significant comparisons between each pair of groups (*p* < 0.05 in dark red and *p* < 0.001 in bright red).

#### 3.3.1. Respiration-to-cardiac coupling functions

The coupling from respiration to the cardiac rhythm, considered to be responsible for respiratory sinus arrhythmia (RSA), has been investigated both centrally and when propagated in the blood flow.

*Central network:* For Y subjects, the coupling function is roughly sinusoidal. The golden arrow ${\bar{\rho }}_{R,C}$ in the polar plot has close-to-100% modulus and its phase is approximately π/2: it can be seen from analysis of the distribution of the gray arrows that the whole group exhibits similarly shifted forms of coupling. For A and ATH subjects, the shapes and phases of the average forms shown in Figures 6B,C are similar: they resemble what has been discussed for Y subjects, but with smaller $\left|{\bar{\rho }}_{R,C}\right|$ and a larger number of phase-outliers. Figure 6D shows the results for surrogates. It can be seen that the amplitude of the coupling for surrogates is negligible when compared to the real cases, and that the phases of surrogates 〈ρ_*R, C*_〉 are scattered around the 2π plane with very variable |ρ_*R, C*_|, resulting in a significantly smaller $\left|{\bar{\rho }}_{R,C}\right|$.

The median values of σ_*R, C*_ are given in Figure 8A. In the case of the central network, the strength of the direct coupling exerted on the cardiac component by the respiratory mode differs significantly from that of the corresponding surrogates, for all groups (*p* < 10^−8^). For Y subjects, σ_*R, C*_ differs significantly from that of the other groups (*p* < 10^−6^), while the A and ATH subjects show overlapping medians (*p* > 0.6). The same significance pattern appears in the similarity box-plots of Figure 8B: only the comparison between A and ATH groups is not significant. For the similarity, the distribution of surrogates is spread along the 0-100% axes of |ρ_*R, C*_|, with a median value around 70%, while the distributions computed for all three Y, A, ATH groups, are all narrowly grouped around median values lying above 95%.

*Peripheral network:* Figure 6E shows that the form of coupling discussed for $q_{C}^{R,C}$ is still detectable in $q_{c}^{r,c}$ for the Y group even if with considerably smaller amplitude. The forms of coupling for the A group (Figure 6F) and ATH (Figure 6G) subjects are similar to that obtained from the surrogate data (Figure 6H). The polar plots of Figures 6F,G show that the A group and ATH subjects are characterized by lower moduli of similarity |ρ_*r, c*_|, with phase shifts 〈ρ_*r, c*_〉 scattered all around 2π. The similarity boxplots in Figure 8C show that |ρ_*r, c*_| for the Y subjects is significantly higher (*p* < 0.05) than that for the three other groups (including the “surrogate group”), and that there are no significant differences between those three groups. The coupling strength σ_*r, c*_ for the A and ATH groups does not differ significantly from that obtained from surrogate data. The only significance found was between Y group and surrogate data (see Figure 8A).

#### 3.3.2. Myogenic-to-cardiac coupling functions

The coupling between myogenic and cardiac oscillations was also investigated both centrally and when propagated in the blood flow. The phase of the myogenic oscillations extracted from LDF was used in both cases, while the phase of the cardiac oscillations was extracted from ECG or LDF respectively.

*Central network:* The forms of coupling function in the first row of Figure 7 follow the same trend as the cardio-respiratory ones: in each group—except for the surrogates—a sinusoidal wave clearly propagates along the ϕ_*C*_ axes. The amplitude of the averaged forms decreases from Figures 7A,B), and from Figures 7B,C. The polar plots show that the number of subjects with smaller |ρ_*m, C*_| and scattered 〈ρ_*m, C*_〉 increases with age and especially with hypertension. The values of $\left|{\bar{\rho }}_{m,C}\right|$ obtained from surrogate data shown in Figure 7D are comparatively small, and 〈ρ_*m, C*_〉 is randomly 2π-scattered. The values of σ_*m, C*_ in Figure 8A, for both the A and ATH subjects are indistinguishable from those for surrogate data. However, σ_*m, C*_ for the Y group it is significantly higher than that for each of the other three groups (*p* < 0.05). In contrast, the similarity of forms |ρ_*m, C*_| clearly distinguishes between the ATH and A groups. Again, the box-plots shown in Figure 8D summarize the results: it is evident that the values for Y and A subjects do not differ, while values for ATH subjects do not differ significantly from those obtained from surrogate data.

*Peripheral network:* Figure 7E is qualitatively very similar to Figure 7A, but with a small relative phase-shift between $\langle {\bar{\rho }}_{m,C}\rangle$ and $\langle {\bar{\rho }}_{m,c}\rangle$. The statistical analysis for σ_*m, c*_ and for |ρ_*m, c*_| follows the same trend detected from the central network. The polar plot in the Figure 7F shows that the phases 〈ρ_*m, c*_〉 are clustered around a $\langle {\bar{\rho }}_{m,c}\rangle$ very close to $\langle {\bar{\rho }}_{m,C}\rangle$: from this plot and from the golden boxes in Figure 8E, it can be seen that most of the subjects from the A group preserve a considerable |ρ_*m, c*_| that is not statistically different from the Y group's distribution. For hypertension (Figure 7G), the average form of the coupling shown in Figure 7F is more ragged. The phases 〈ρ_*m, c*_〉 are scattered in the 2π plane, and the coupling between ϕ_*m*_ and ϕ_*c*_ generates forms with small |ρ_*m, c*_|. The boxes of Figure 8E summarize the results, indicating no difference in |ρ_*m, c*_| between Y and A groups, while values for the ATH group are indistinguishable from those obtained from surrogate data.

## 4. Discussion

By analysis of phase coherence and coupling functions we have been able to study how cardiovascular and microvascular dynamical processes change with aging and hypertension using only resting-state recordings, without any need to stimulate or perturb the system.

The significant increase in very slow (≤ 0.02 Hz), respiratory and cardiac oscillations in blood flow with age (Figure 4) could be explained by results obtained in earlier studies which showed that small arterioles are dilated in the elderly (Kelly et al., 1995) and that the diameter of the larger arteries increases together with a decrease in elasticity (Levy, 2001). Hence, the increased vessel radii and decreased vessel elasticity may have resulted in larger oscillations. The striking difference between the A and ATH groups time-averaged wavelet power in the cardiac frequency range shows that cardiac pulses in the ATH group are not restored to age matched normal. This suggests an increased stiffness of arterioles in ATH patients, a defect that persists despite antihypertensive therapy.

The reduction in 0.1 Hz interval coherence seen in the ATH group (Figure 5) indicates an additional cardiovascular system abnormality that is not restored by antihypertensive treatment to age matched normal. The results appear to imply a progressive impairment with age of the underlying mechanisms of coordination between cardiac and vascular activity, and with even greater impairment in hypertension.

The lack of significant 0.1 Hz interval coherence between R-R and SBF in the ATH group suggests local changes of the skin microvasculature, an inference that is supported by an additional finding: a loss of significant 0.1 Hz interval coherence between blood flow measured on different sites (not shown). This may perhaps be an indication of impaired myogenic activity observed via the blood flow signal. This explanation is consistent with the reported increase of stiffness and basic myogenic tone in the arteries of hypertensive patients (Feihl et al., 2006; Yannoutsos et al., 2014), which may also explain the decrease in the amplitude of cardiac oscillations in the blood flow of the ATH group (Figure 4). This abnormality is not restored to aged matched normal by treatment.

Coupling functions provide additional insight into the changes that occur with aging and hypertension. For the central network, the mechanism of RSA is captured by the coupling functions. The sinusoidally-shaped wave propagating along the respiration phase axes ϕ_*R*_ in Figure 6A indicates that the coupling between ϕ_*R*_ and ϕ_*C*_ with the cardiac rhythm depends on the phase ϕ_*R*_: namely, the heart rate accelerates on the second part of the respiratory cycle i.e., after inhaling ($q_{C}^{R,C}$ <0), and decelerates during the initial part of the cycle, i.e., after exhaling ($q_{C}^{R,C}$>0). These results are consistent with earlier work (Iatsenko et al., 2013; Kralemann et al., 2013). Furthermore, the polar plots give additional insight into the group dynamics. The comparison between groups in Figures 6A–C suggests that RSA weakens but is nonetheless preserved in aged subjects. Treatment for hypertension does not seem to influence this phenomenon.

For the peripheral network, the difference in coupling between respiratory and cardiac waves between the groups of young and aged subjects becomes more evident. Within this network, the phase shift displayed by the form of coupling for young subjects (Figures 6A,E) reflects the time-delay that the respiration wave undergoes during its propagation to the peripheral vascular network, Because the distribution of |ρ_*r, c*_| for the aged groups is not significantly different from that of the surrogates, we conclude that the interaction between the phases ϕ_*r*_ and ϕ_*c*_ in the vascular bed weakens with aging. This result is not dependent on the propagation of the waves themselves: the investigation of spectral power shown in Figure 4 revealed that both the respiratory and cardiac oscillations are stronger in healthy aged subjects than in the young group (*p* < 0.05). Aging-related loss of tone in the walls of big vessels is thought to play a role by offering less resistance to blood-flow and therefore easing the propagation of centrally-generated waves (Levy, 2001).

The most interesting outcomes of the study are related to the myogenic oscillation. The reduced coherence of the myogenic interval seen in the ATH group (Figures 5B,C) indicates a cardiovascular system abnormality that is not restored by antihypertensive treatment. The results appear to imply a progressive impairment with age of the underlying mechanisms of coordination between cardiac and vascular activity, and with even more impairment in hypertension. Antihypertensive treatment is evidently unable to correct this defect; we found no evidence to suggest that the medications listed in Table A1 differ in efficacy in this respect. The treatments were diverse, however, and the number of subjects under exactly the same treatment regimens were too small to allow for a reliable statistical comparison.

To get deeper insight into this “asynchrony,” myogenic-to-cardiac couplings were analyzed and studied here for the first time. For young subjects, the coupling strength σ_*m, C*_ of the central network is significantly higher than that for the other groups. This result indicates that the myogenic activity of the skin microvessels shares functional properties with those of the cardiac muscle, and it confirms that the coupling between myogenic activity detected in microvasculature and cardiac activity fades with aging. Box-plots in Figure 8D show how both Y and A, but not the ATH group, have a similarity modulus significantly higher than surrogates.

Analysis of the peripheral network for the myogenic-cardiac interaction generated similar results. There was an even clearer difference between the moduli of similarity obtained from A and ATH subjects. Box-plots for the similarity of the forms in Figure 8E cluster the subjects into two statistically distinct groups: Y with A, and ATH with surrogates.

It had already been shown that antihypertensive medication does not necessarily improve endothelial function (Ghiadoni et al., 2003). Moreover an impaired efficiency of myogenic activity within the hypertensive vascular system was also suggested by an earlier study, which did not improve with anti-hypertensive treatment (Rossi et al., 2006). Results for the similarity modulus |ρ| demonstrate that myogenic and cardiac oscillations are less strongly coupled in hypertensive than in healthy aged subjects, despite the treatment. This highlights how the comparative similarity of forms can reveal characteristics of the coupling mechanism that would remain undetected by investigations just based on coupling strength. Counter-intuitively, the myogenic spectral power (box-plots in Figure 4) was found to be significantly higher in hypertensive than in young subjects. Similarly to what was discussed for the case of respiration, this outcome supports the theory that what is affected is not the magnitude of the oscillation, but its capacity to adjust to the dynamical interactions to which it is being subjected.

One can expand the analyses presented here, and the phases of the three oscillatory components can also be extracted from other signals, if simultaneously recorded. One such candidate is a signal providing information proportional to the blood pressure, rather than to blood flow as used in this study. For example, the signal of finger pulse photoplethysmography (PPG, see e.g., Allen, 2007) was simultaneously recorded in all our subjects and is therefore available together with the other signals. The changes in finger volume result from the involvement of arterioles, as well as the microvasculature, and hence the myogenic contribution comes on average from larger vessels than those recorded by the LDF. To verify whether the observed difference in coherence between the R-R intervals and the blood flow at the myogenic interval between A and ATH groups also exists when larger vessels are included, the same analysis as that presented in Figure 5B was performed, but using the PPG signal. No statistically significant differences were observed between the two groups (see **Appendix II**), which can be taken to indicate that the blood pressure control mechanisms related to smooth muscle cells are probably restored by the current antihypertensive treatment. This further demonstrates that it is the endothelial involvement (Furchgott and Zawadzki, 1980) that is still impaired, as this is dominant in the microvasculature. These results are in agreement with an earlier study (Ebert et al., 1992) where, based on recordings of muscle sympathetic nerve activity (MSNA), it was shown that, although the parasympathetic component of the arterial baroreflex becomes impaired with advancing age, the sympathetic component can be well maintained in healthy individuals even into the seventh decade. The methodology presented here can thus be further used to investigate coherences and couplings from any signals recorded simultaneously from the cardiovascular system. Note, however, that special care is needed: a minimum length of recording is required; and the phase of the oscillations should be extracted with sufficient precision for the calculations to be meaningful.

A difficulty in doing research on treated hypertensive patients is the large range of different drugs, each with its own separate mode of action, that one may encounter. Indeed, as shown in Table A2, a wide variety of drugs was used to treat the subjects included in our study: their inclusion was based on the fact that their hypertensive treatment has been individually optimized for maximal success. So our study does not make it possible to find out which of the drugs are less, or more, effective. Nonetheless, in a cohort of patients with optimally treated hypertension, according to the current doctrines, we have shown that there is still residual microvascular impairment. The same methodology can clearly be used to evaluate the effect of individual drugs, or for a longer-term follow up of a treatment for hypertension, and may help in the development of new medications.

In conclusion, by investigating the deterministic properties of the HRV signal together with simultaneously recorded respiration and microvascular blood flow signals, and by extracting time-dependent parameters, we have gained insights that are clinically relevant to studies of aging and hypertension. While cardiorespiratory couplings and interactions in general have been studied previously, here we have investigated for the first time phase coherence and coupling between myogenic activity and cardiac and respiratory oscillations. The significant impairment of coherence within the myogenic interval of the ATH group as recorded in the microvasculature seems to imply that some of the current treatments for essential hypertension fail to restore microvascular regulation. Similarly, treated hypertensive subjects differ from the healthy control group of the same age in terms of the coupling between cardiac and myogenic oscillations within the peripheral network. Thus hypertension affects the myogenic microvascular structure by uncoupling the system of oscillators of which it is composed. It is clear that current anti-hypertensive treatments, while successfully controlling blood pressure, do not restore microvascular function.

## Ethics statement

This study was carried out in accordance with the recommendations of UK Northwest Research Ethics Committee with written informed consent from all subjects. All subjects gave written informed consent in accordance with the Declaration of Helsinki. The protocol was approved by the UK Northwest Research Ethics Committee.

## Author contributions

VT completed the coupling functions analysis with advice and supervision from TS created figures, and drafted the manuscript. DI completed the phase coherence analysis, created figures, and drafted the associated text. AB measured and analyzed data. AEB helped to recruit the hypertensive patients, measured data and did preliminary analysis. AG recruited and selected the hypertensive patients. PM helped to supervise the study and contributed to writing the manuscript. PC provided clinical support and contributed to the interpretation of the results. AS conceived, planned and supervised the study, and contributed to the development of the algorithms and to the writing of the manuscript. All authors discussed the results and contributed to the editing of the manuscript.

### Conflict of interest statement

The authors declare that the research was conducted in the absence of any commercial or financial relationships that could be construed as a potential conflict of interest.

## Figures and Tables

**Figure A1:**
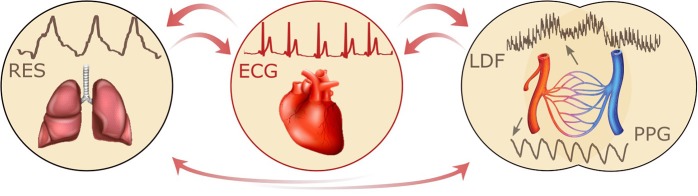
Schematic representation of the interactions between respiratory, cardiac and vascular activity, together with the corresponding recordings: respiratory effort signal (RES), electrocardiogram (ECG), laser Doppler flowmetry (LDF) and pulse plethysmography (PPG). In the resting state, the concentration of red blood cells can be considered constant, and so a Doppler shift in the velocity signal provides a measure of microvascular flow. In contrast, the finger PPG provides a measure of changes in arterial volume proportional to changes in arterial blood pressure.

**Figure A2:**
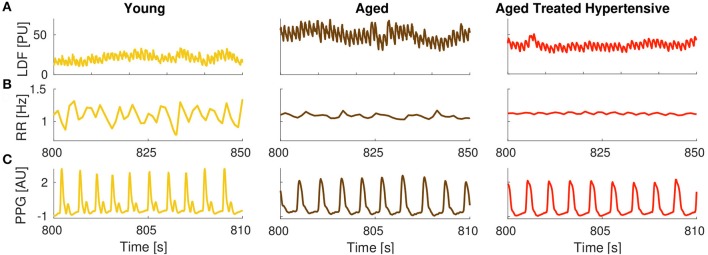
Typical time series: **(A)** Skin blood flow measured by the LDF (also referred to as SBF); **(B)** RR interval (RRI); and **(C)** PPG signals from each group of subjects. PU stands for perfusion units, and AU for arbitrary units.

## Appendix I – parameters for patients and healthy subjects

**Table A1:** Summary of the hypertension-related parameters and of the medication being taken by the ATH group.

**Parameter**	**Mean ± SD**	**Medications taken**	**Number of subjects**
Age (years)	70.3 ± 6.7	Beta-blockers	10
Systolic pressure (mmHg)	138.8 ± 16.4	ACE inhibitors	10
Ankle brachial pressure index	1.08 ± 0.09	Angiotensin receptor blockers	4
Total cholesterol (mmol/l)	4.26 ± 1.22	Calcium channel blockers	9
HDL cholesterol (mmol/l)	1.36 ± 0.38	Diuretics	8
LDL cholesterol (mmol/l)	2.32 ± 0.99	Statins	16
Triglycerides (mmol/l)	1.31 ± 0.53	Aspirin	12
hs-CRP (mg/l)	2.62 ± 2.03		
Capillary refill time (s)	2.5 ±0.6		
Height (m)	1.68 ± 0.10		
Weight (kg)	77.6 ± 16.5		
Body mass index (kg/m^2^)	27.2 ± 3.9		
Time since diagnosis (years)	10.0 ± 6.2		

## Appendix II – effect of hypertension on PPG dynamics

To further investigate the signatures of aging and treated hypertension in cardio-vascular regulation, we describe in this Appendix results obtained by phase-coherence analysis of pulse plethysmography (PPG) time series. The analyses and comparisons between groups were performed in the same way as for the laser Doppler flowmetry (LDF) time series discussed in the main text.

Figure A1 illustrates schematically how both the LDF and the PPG data reflect the oscillations of the circulatory activity associated with different vascular levels, i.e., those of the capillary bed for LDF and those of the bigger vessels for PPG. The PPG was measured by a Finapress device (Omboni et al., 1993).

The different nature of the two signals is evident in Figure A2, where LDF, RR-interval and PPG time series are shown for a typical subject from each group: young (Y) in gold, aged (A) in brown, aged treated hypertensive (ATH) in red.

The coherence analyses are compared in Figure A3, with RR-PPG in panels **(A,B)** and RR-LDF in **(C,D)**. The results plotted in Figures A3B,D show that for all groups there is significant coherence (i.e., above the significance threshold) in the coherence of both PPG and LDF and the RR intervals in the respiratory and myogenic intervals.

For the PPG-RR coherence shown in Figure A3B, there is a significant difference between the Y and A (gold shading) and the Y and ATH groups (gray shading), but no significant difference between A and ATH. The similarity between A and ATH coherence, when compared to the Y pattern, also emerges from the time-localized group average in Figure A3A.

In contrast, the coherence between LDF and RR appears to decline with age, and it nearly disappears in treated hypertension, as illustrated in Figure A3D. Moreover, the comparison between A and ATH, which cannot be distinguished by the PPG-RR coherence, exhibits significant differences within the myogenic interval, as discussed in the main text. Figure A3C presents the group-averaged time-localized coherence between RR intervals and blood flow.

These findings suggest that the LDF dynamics, which is a measure of the oscillatory patterns of capillary flow, is more sensitive to the effect of treated hypertension than PPG dynamics, probably because the latter reflects oscillations within bigger vessels.

**Table A2:** Individual characteristics of the ATH group: Y, Yes; N, No; NA, Not available. The Yes boxes are shaded for easier visual appraisal.

**Age**	**Sex**	**Blood Pressure (mmHg)**	**Years since diagnosis**	**RR-PPG myogenic coherence**	**RR-LDF myogenic coherence**	**Beta-blockers**	**ACE inhibitors**	**Angiotensin receptor blockers**	**Calcium channel blockers**	**Diuretics**	**Statins**	**Aspirin**
70	F	NA	16	Y	Y	Y	N	N	N	N	Y	N
69	M	NA	7	Y	Y	N	Y	N	N	N	Y	Y
71	M	162	10	Y	N	Y	Y	N	N	Y	Y	Y
66	F	150	12	Y	Y	N	Y	N	N	Y	Y	Y
60	F	171^*^	9	Y	Y	N	N	N	N	N	N	Y
75	F	145	6	Y	N	Y	N	N	Y	Y	Y	Y
70	M	131	7	Y	N	Y	N	N	Y	N	Y	Y
79	M	120	13	Y	N	N	Y	N	N	N	N	N
74	F	145	14	Y	N	N	Y	N	N	Y	Y	Y
80	F	138	NA	Y	N	Y	N	Y	Y	N	Y	Y
68	M	115	2	Y	N	N	Y	N	N	N	Y	Y
64	M	155	8	Y	Y	Y	Y	N	N	N	Y	N
68	M	125	6	Y	N	Y	N	Y	N	N	Y	N
84	F	145	28	Y	N	Y	N	N	Y	N	Y	N
59	M	125	6	Y	N	N	N	Y	N	N	Y	N
69	F	128	8	Y	N	N	Y	N	N	N	N	N
83	M	140	17	Y	Y	N	N	N	Y	Y	Y	Y
72	M	128	12	Y	Y	Y	N	N	Y	Y	Y	Y
65	M	120	2	Y	Y	N	Y	N	Y	N	Y	Y
70	F	120	NA	Y	Y	Y	Y	N	Y	Y	N	N
65	M	155	14	Y	Y	N	N	Y	N	Y	N	N
65	F	160	2	Y	Y	N	N	N	Y	N	N	N

* *A typical “white coat hypertensive,” her readings ranged from 171 mmHg (on the day of measurements), through 150 mmHg with a doctor, down to 129 mmHg with a nurse*.
